# Constructing Supported Cell Membranes with Controllable Orientation

**DOI:** 10.1038/s41598-019-39075-8

**Published:** 2019-02-26

**Authors:** Shao-Wei Lyu, Jou-Fang Wang, Ling Chao

**Affiliations:** Department of Chemical Engineering, National Taiwan University, Taipei, Taiwan

## Abstract

Membrane proteins play important roles in various cellular processes. Methods that can retain their structure and membrane topology information during their characterization are desirable for understanding their structure-function behavior. Here, we use giant plasma membrane vesicles (GPMVs) to form the supported cell membrane and develop a blotting method to control the orientation of the deposited cell membrane in order to study membrane proteins from either the extracellular or the cytoplasmic sides. We show that the membrane orientation can be retained in the directly-deposited membrane and the deposited membrane on mica can be blotted onto glass to reverse the membrane orientation. We used Aquaporin 3 (AQP3), an abundant native transmembrane protein in Hela cells, as a target to examine the cell membrane orientation in the directly-deposited and reversed membrane platforms. The immunostaining of antibodies targeting either the cyto-domain or ecto-domain of AQP3 shows that the intracellular side of the cell membrane faced the bulk aqueous environment when the GPMVs spontaneously ruptured on the support and that the membrane orientation was reversed after blotting. With this blotting method, we can thus control the orientation of the supported cell membrane to study membrane protein functions and structures from either side of the cell plasma membrane.

## Introduction

Membrane proteins play an important role in various cellular processes^1,2^. Due to their amphiphilic properties, they can easily denature and lose their functions when they are purified from cells for characterization^3–5^. In order to maintain membrane protein structures and functions, many studies have tried to study membrane proteins through the use of lipid membranes, including reconstituting the proteins into artificial lipid membranes^3,6–10^ or preparing membrane vesicles directly from cells^11–16^.

When membrane proteins are studied and characterized, it is important not only that their structures be kept intact but also that their natural topologies be maintained. Many membrane proteins have different cytoplasmic and extracellular domains which are in charge of different functions^17,18^. As such, the question of whether the natural topologies of membrane proteins can be maintained or controlled during their characterization is an important issue. Previous studies have shown that it is challenging to control the insertion orientation of membrane proteins while reconstituting the proteins into artificial lipid membranes^6,7,17,18^. Therefore, many studies have obtained cell membrane vesicles directly from cells as these membrane vesicles should preserve the native orientation of membrane proteins^12–16,19^.

Several studies have further deposited such cell membrane vesicles on a solid substrate to form supported membranes^13–16,19–21^ for characterization purposes since the planar geometry of such supported membranes is compatible with many surface characterization tools^21–23^. However, some studies have shown that the outer leaflets of the cell membrane vesicles faced toward the bulk solution (that is, the outer leaflets were still facing outside-out) after they ruptured on the support^8–10,14,16^, whereas other studies have shown the opposite result – that is, that the inner leaflets faced toward the bulk solution^13,20^. These different results might be due to the different membrane vesicle sizes used, as suggested by Tutus *et al*.^9^, or to the different preparation methods used to prepare the membrane vesicles. In any case, these different observations suggest that even if a supported cell membrane can retain its native orientation, it is still unclear how to control which side of the membrane faces toward the bulk solution.

In this study, we developed a blotting method to reverse the orientation of the deposited membrane from giant plasma membrane vesicles (GPMVs) in order to study membrane proteins from either the extracellular side or the cytoplasmic side. We used the immunostaining of antibodies targeting either the cyto-domain or ecto-domain of Aquaporin 3 (AQP3), a native membrane protein in the used cell, to examine the membrane orientation of the derived GPMVs, the directly-deposited plasma membranes, and the blotted plasma membranes. The results show that we can construct cell membrane platforms with controllable membrane orientation.

## Results and Discussions

### Membrane orientation of giant plasma membrane vesicles (GPMVs) from cells

**Figure 1:**
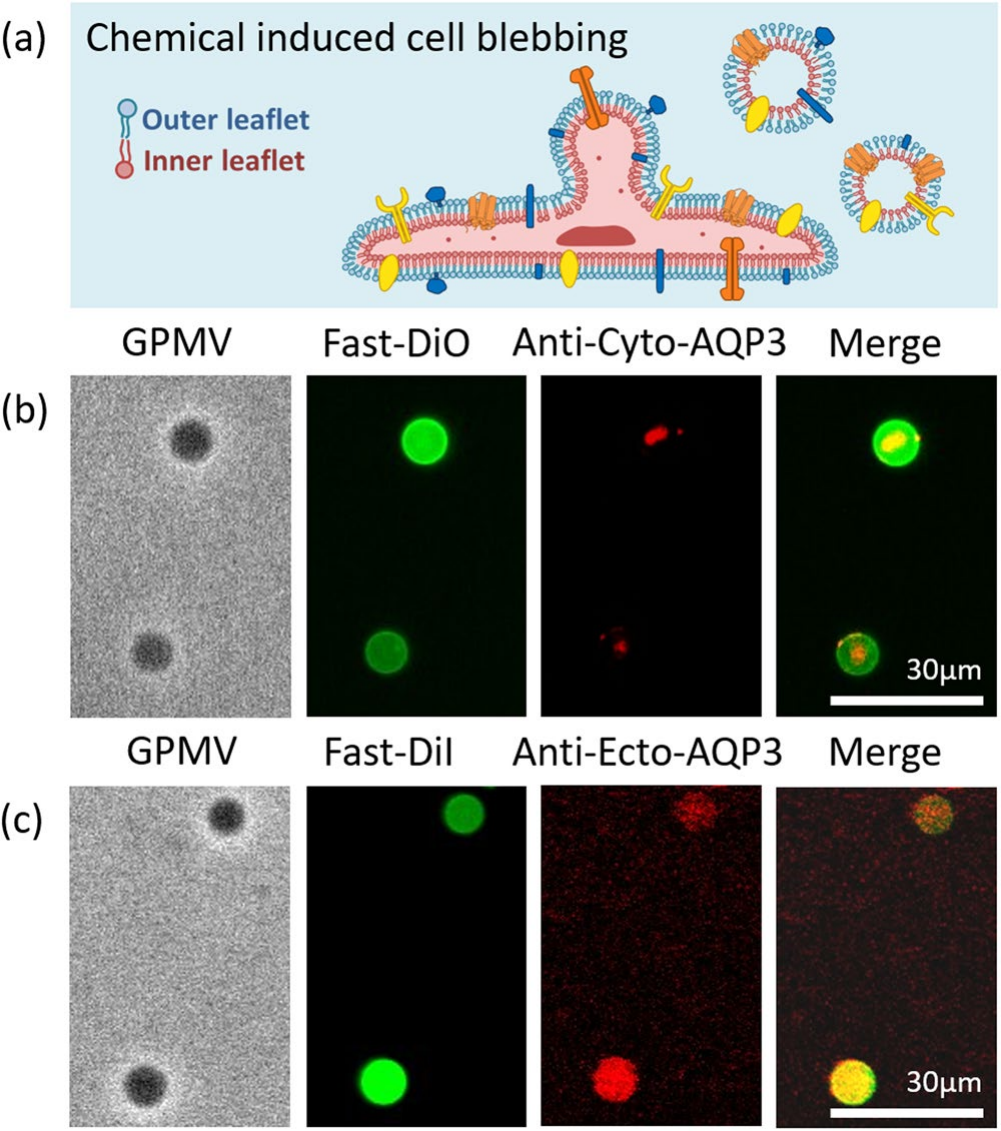
(**a**) Illustration of the orientation of GPMVs derived from a cell. (**b**) Immunostaining of GPMVs with antibodies targeting the cyto-domain of AQP3. (**c**) Immunostaining of GPMVs with antibodies targeting the ecto-domain of AQP3. Left panel: bright field images. Middle-left panel: fluorescence images showing the lipid probes. Middle-right panel: fluorescence images showing the antibodies. Right panel: merged images of lipid probe and antibody images. Note that the fluorescence images in (**c**) have fake colors for comparison with those in (**b**). GPMVs are stained with different lipid dyes because the two commercial antibodies have fluorophores illuminating at different wavelengths.

Figure 1 shows the GPMVs directly blebbed out from the plasma membrane via the chemical blebbing method^12^. We hypothesized that the chemicals cause defects in the cytoskeleton, and the osmotic pressure difference inside and outside the cell causes the plasma membrane to bleb out. Therefore, the GPMVs should have the same membrane orientation as the original cell, and the ecto-domain of proteins should face out as shown in Fig. 1(a).

In this study, we examined the membrane orientation of the GPMVs by immunostaining of an asymmetric transmembrane protein, Aquaporin 3 (AQP3). We used the cell membrane from Hela cells without any gene overexpression or modification, and the native AQP3 in the membrane should be in their native topology and therefore we use the known topological information of AQP3 to postulate the membrane orientation. We chose AQP3 as the target since it is an abundant membrane protein in non-transfected Hela cells, and both the antibodies targeting to the cyto-domain and ecto-domain of AQP3 were validated for immunofluorescence (IF) in cells. The left and middle-left panels in Fig. 1(b,c) are the bright images and the fluorescence images of the GPMVs which were labeled with fluorescent lipid dyes, Fast-DiO or Fast-DiI. The middle-right images show that anti-ecto-AQP3 bound to the entire GPMV membrane surface, while anti-cyto-AQP3 seems to bind to the inner part of the GPMV. The result supports that the derived GPMVs have their extracellular side facing-out, same as the natural membrane orientation of a cell.

### Blotting to reverse the orientation of deposited GPMV membranes

**Figure 2:**
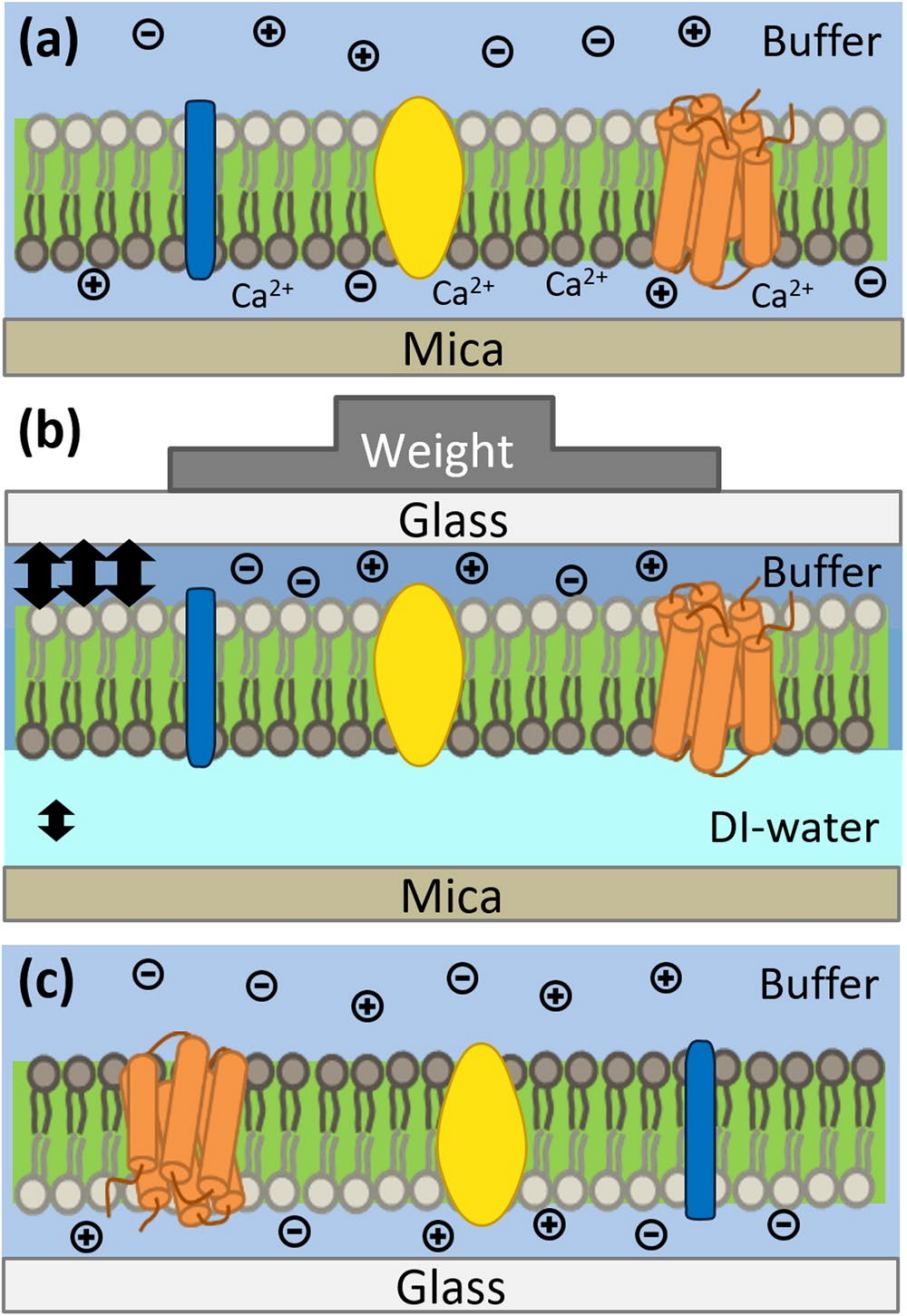
Illustration of the weight blotting process of membrane pre-formed on mica and transferred onto the glass. The lipid leaflet with dark gray color represents the lipid leaflet next to the formation support and the one with bright gray color represents the leaflet facing to the bulk solution when the directly-deposited membrane is formed.

We have previously shown that we can rupture GPMVs onto a glass surface to form supported plasma membranes, and the immunostaining results suggested that the supported plasma membranes have their intracellular sides facing the bulk solution^19^. In order to study either side of a supported plasma membrane, we intended to develop a blotting technique to reverse the membrane orientation. To blot the supported membrane to another support, the blotting support needs to have a higher affinity with the membrane than the formation support (Fig. 2). However, if the affinity is too strong, the membrane may stick to the blotting support with no water layer between the membrane and the support, leading to the loss of fluidity.

Here, we used mica and glass as our formation support and blotting support since previous studies have shown that supported lipid bilayers can form well and possess fluidity on these conventional supports. One previous study has shown that the presence of calcium ion is more critical for the formation of supported membranes on mica but not on glass^24^. This observation suggests that the removal of calcium ions may reduce the affinity between the membrane and mica while the affinity between the membrane and glass can remain. Therefore, we first deposited GPMVs on mica to form the supported plasma membrane in the presence of calcium ions and then removed the calcium ions before blotting the plasma membrane to a glass support. In addition, since both the mica surface and the plasma membrane are negatively charged, reducing the ionic strength of the water layer between mica and the membrane can increase the electrostatic repulsion between the mica support and the membrane. Therefore, to reduce the affinity between mica and the membrane, we immersed the supported membrane on mica in DI-water for 30 min before the blotting in order to reduce not only the number of calcium ions but also the ionic strength of the water layer between the mica surface and the membrane. Right before the blotting process, we replaced the DI-water above the membrane with a PBS buffer with physiological ionic strength in order to reduce the electrostatic repulsion between the incoming negatively charged glass surface and the negatively charged membrane. Since we assume that it takes time for the ions to significantly invade into the thin water layer between mica and the membrane, the low calcium ion concentration and low ionic strength maintained during the blotting process could allow the mica to have a relatively low affinity with the membrane compared to the blotting glass. This decreased affinity of the membrane with the formation support and the increased affinity with the blotting support could lead to effective blotting.

### Immunostaining to examine the membrane orientation of the supported plasma membrane platforms

**Figure 3:**
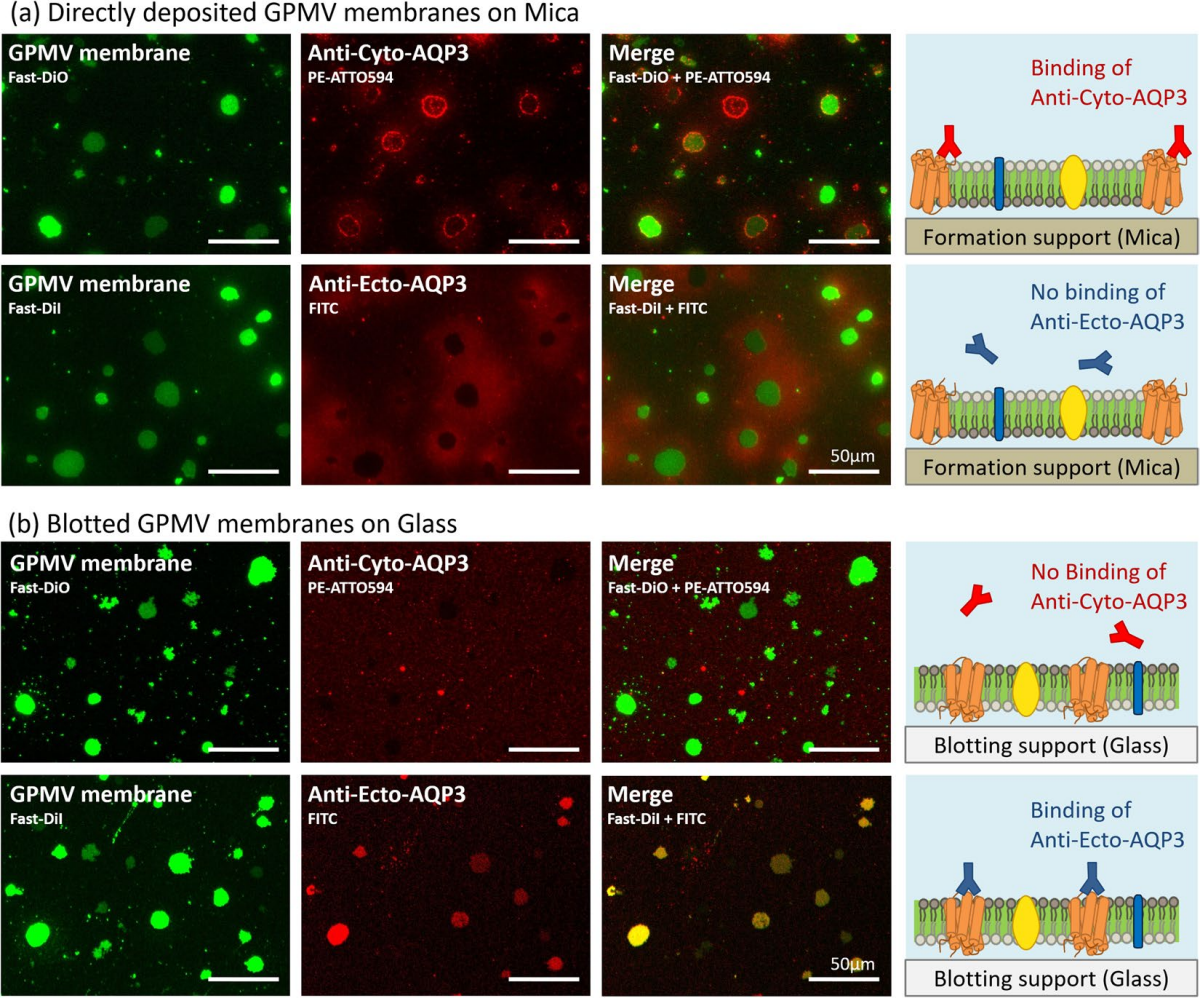
Immunostaining results of a transmembrane protein, Aquaporin 3 (AQP3), in the supported plasma membrane. (**a**) The membrane patches directly formed on mica. (**b**) The membrane patches blotted onto the glass. The lipid leaflet with dark gray color represents the lipid leaflet next to the formation support and the one with bright gray color represents the leaflet away from the formation support when the directly-deposited membrane is formed.

To examine the topologies of AQP3 in the directly-deposited membrane and the blotted membrane, we used anti-cyto-AQP3 and anti-ecto-AQP3, which bind to the cyto-domain and ecto-domain of AQP3, respectively (see Supplementary Fig. S1). Figure 3(a) shows the positive immunostaining of anti-cyto-AQP3 and negative immunostaining of anti-ecto-AQP3 in the directly-deposited membrane platform, suggesting that the intracellular side of the membrane faced toward the bulk solution and the extracellular side faced toward the support. After the blotting (Fig. 3(b)), the positive staining of anti-ecto-AQP3 and the negative staining of anti-cyto-AQP3 suggest that the extracellular side of the membrane faced toward the bulk solution and the cytoplasmic side faced toward the support. More importantly, the comparison between the lipid probe images and the antibody images shows that almost all of the patches showed the same orientation.

In order to eliminate the possibilities that these two antibodies non-specifically bind to lipids in the lipid membrane, we prepared artificial supported lipid bilayers composed of 1,2-dioleoyl-sn-glycero-3-phosphocholine (DOPC) as typical lipid membrane controls. The results show that the nonspecific binding of the two antibodies to the lipid membrane is weak (see Supplementary Fig. S2). Since we used native Hela cell membranes, it is difficult to exclude the possibilities that the antibodies non-specifically bind to the other contents in the cellular membrane patches. However, in our results, the anti-cyto-AQP3 has positive staining only to the directly-deposited membrane but not to the blotted membrane. If the antibody can easily bind to the other contents in the membrane patches, it should bind to not only the directly deposited membrane but also the blotted membrane, which is not the situation in our results. Similarly, the anti-ecto-AQP3 only has positive staining to the blotted membrane but not to the directly-deposited membrane, suggesting that the anti-ecto-AQP3 does not significantly bind to the other contents in the membrane patches.

We noticed that a significant amount of anti-cyto-AQP3 bound to the boundaries of the directly-deposited membrane patches (Fig. 3(a)). We hypothesized that the phenomenon is due to the non-specific binding of anti-cyto-AQP3 to the patch boundary since we did not observe the bright-boundary situation when another antibody targeting to the cyto-domain of glucose transporter GLUT1 was used (see Supplementary Fig. S3). GLUT1 is also an abundant membrane protein in Hela cells and the positive immunostaining of anti-cyto-GLUT1 in the directly-deposited membrane patches, supporting that the intracellular side of the membrane faced toward the bulk solution. However, we did not find immunofluorescence-validated antibodies targeting the ecto-domain of GLUT1. Therefore, in order to show the immunostaining of the same membrane protein, we displayed the results of anti-cyto-AQP3 for the comparison with the anti-ecto-AQP3 results in spite of the potential non-specific binding to the patch boundary.

Another observation is that both anti-cyto-AQP3 and anti-ecto-AQP3 bind to the surroundings of the directly-deposited membrane patches (Fig. 3(a)). The merged image of the lipid probe image and the antibody image clearly shows that the antibody binding occurred in the concentric-circle-region outside the membrane patches. Since a previous study has shown that numerous cytoplasmic contents can be included in GPMVs^25,26^, we hypothesized that this phenomenon could be due to the non-specific binding of the antibodies to the contents bursting from the GPMVs when they ruptured. Since both anti-cyto-AQP3 and anti-ecto-AQP3 are polyclonal antibodies, they could easily bind in a non-specific manner to many cytosolic contents. On the other hand, we did not observe these concentric-circle-regions when we stained the blotted membranes (Fig. 3(b)), probably because the bursting contents strongly stuck to the formation support and were not easily blotted to the blotting support.

### Membrane fluidity and antibody fluidity in the directly-deposited and blotted membranes

**Figure 4:**
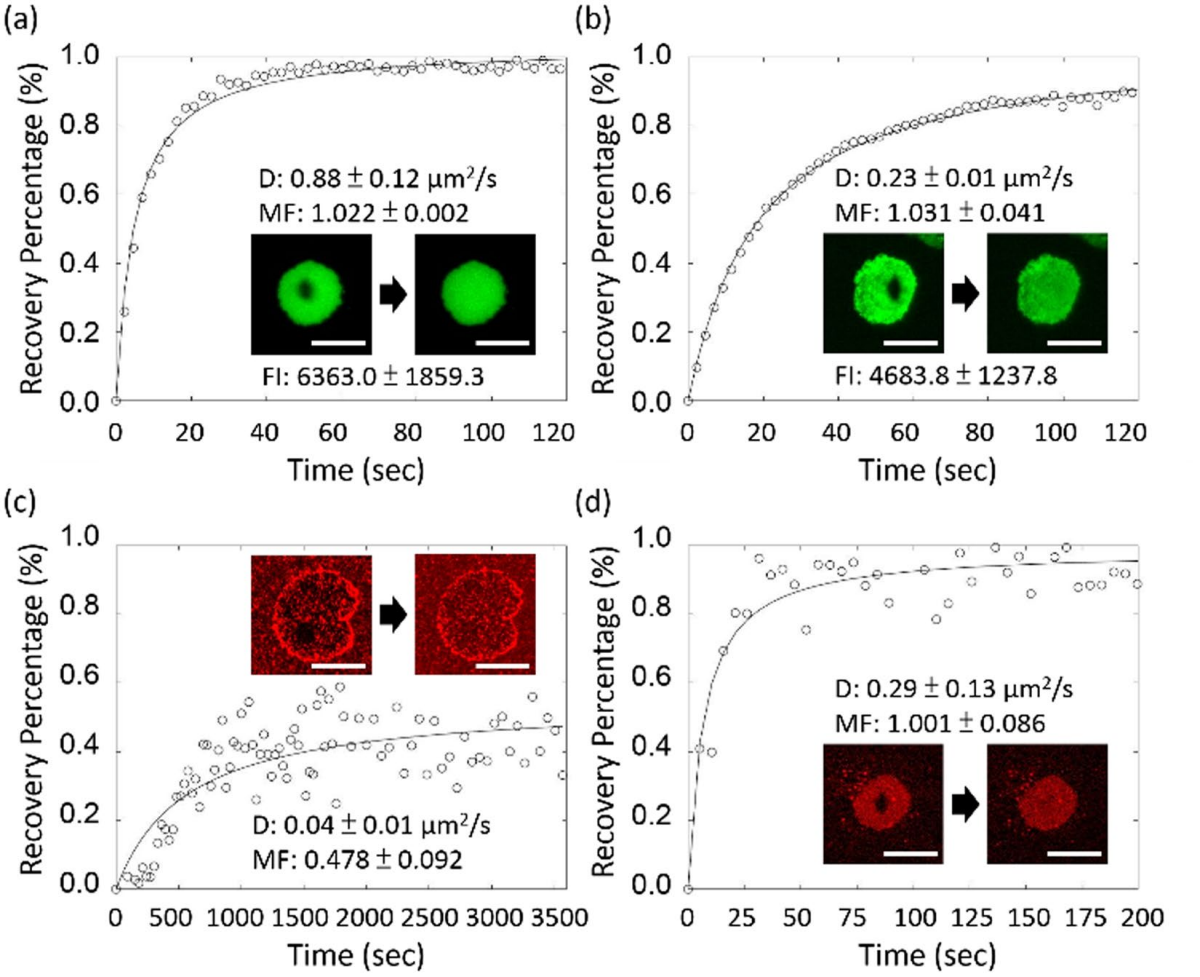
FRAP measurement and fluorescence intensity analysis of (**a,b**) lipid probes in the directly-deposited and blotted membranes, respectively. (**c**) anti-cyto-AQP3 in the directly-deposited membrane; and (**d**) anti-ecto-AQP3 in the blotted membrane. Scale bar: 30 μm. (D: diffusivity. MF: mobile fraction. FI: fluorescence intensity). Data are represented as means with standard deviations (n = 3 for D and MF; n = 12 for FI).

The fluorescence intensity and the diffusivity of the blotted membrane remained high (Fig. 4(b)), suggesting that most of the membrane was successfully transferred from mica to the glass support. However, the quantitative comparison showed that the averaged intensity of the blotted membrane was lower than that of the directly-deposited one as shown in Fig. 4(a,b). More importantly, we measured the lipid fluidity and the antibody fluidity of the directly-deposited and blotted membranes by fluorescence recovery after photobleaching (FRAP). We found that the lipid diffusivity of the blotted membrane on glass (D = 0.23 μm^2^/s) was slower than that of the directly-deposited one on mica (D = 0.88 μm^2^/s). The slower diffusivity indicated that there were some defects in the blotted membrane. However, the mobile fractions of the blotted membranes can still reach one, suggesting that the membrane inside a blotted patch was still connected and remained some integrity. More importantly, Fig. 4(c,d) showed that the fluorescence from both anti-cyto-AQP3 in the directly deposited membrane and anti-ecto-AQP3 in the blotted membrane can recover after photobleached by a laser beam, indicating that AQP3 are mobile in our membrane platforms. Interestingly, the FRAP results showed that the diffusivity of fluorescent anti-ecto-AQP3 in the blotted membrane was larger than that of fluorescent anti-cyto-AQP3 in the directly deposited membrane. It could be attributed to the reason that the extracellular domain is larger than the cytoplasmic domain as suggested by Aquaporin 4^27^ which has known crystal structure and is in the same family as AQP3. The larger protruding part could have more frictions with the support in the directly-deposited membrane, leading to the lower diffusivity.

**Figure 5:**
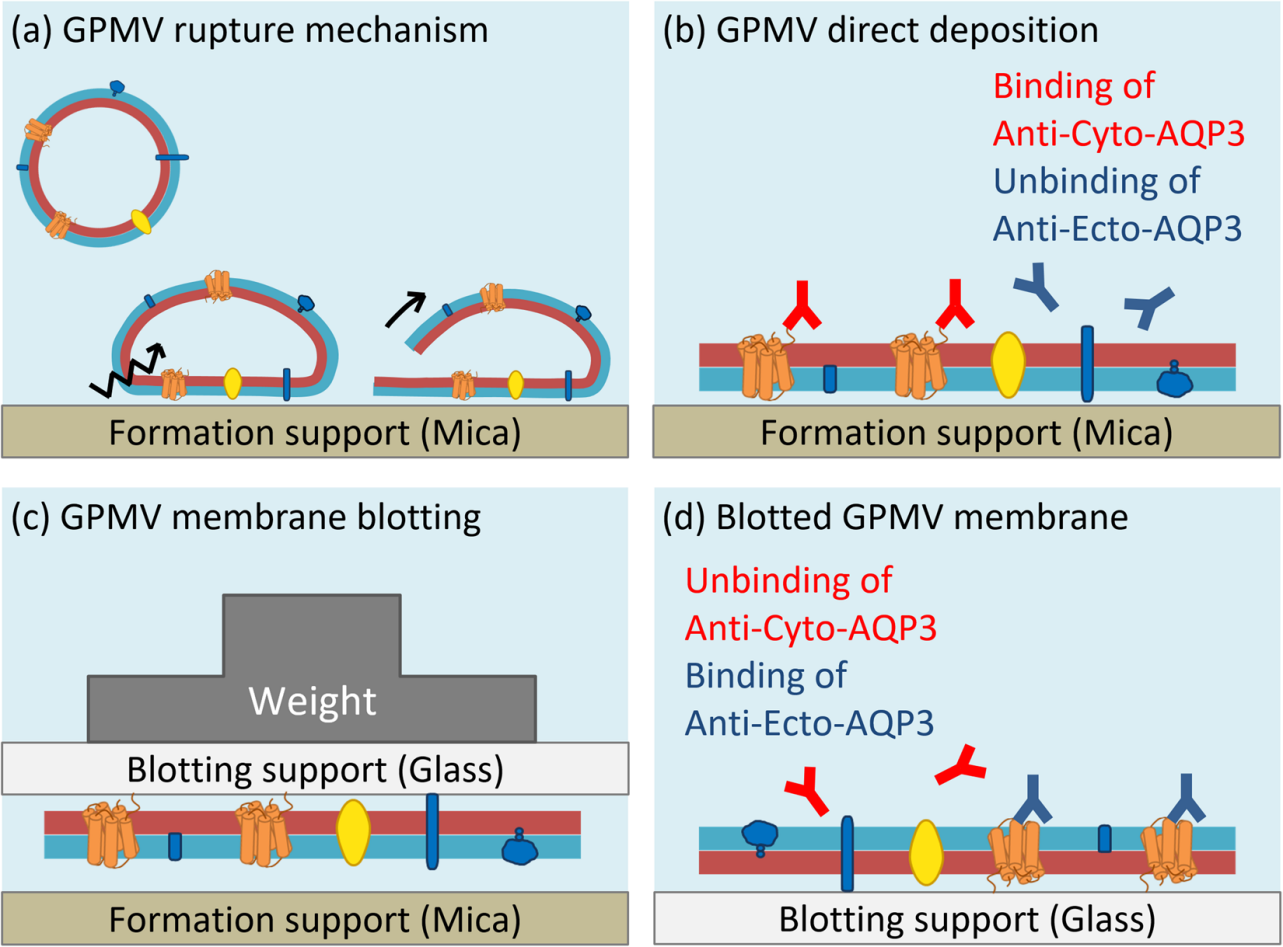
Illustration of membrane orientations of GPMVs derived from cells, the supported plasma membrane formed by the rupture of GPMVs, and the blotted membrane. The blue color span represents the extracellular leaflet and the red color span represents the intracellular leaflet of the cell plasma membrane.

The illustration in Fig. 5 further summarizes the membrane orientations of GPMVs derived from cells, the supported plasma membrane formed by the rupture of GPMVs and the blotted membrane. Our results show that the rupture of a vesicle with a size of tens of microns on a glass or mica surface has its intracellular side facing toward the bulk solution. The result is similar to the rupture situation of a giant unilamellar vesicle (GUV) which rupture process has been directly observed by a high speed camera^28^, and the rupture of human red blood cell ghost membranes which cytoplasmic domains are detected by immunostaining^13,20^. However, it is opposite to the results suggested from the previous studies on using proteoliposomes reconstituted with transmembrane proteins^8–10^ or using small native cell membrane vesicles (150 nm to 200 nm in diameter) to form supported membranes^14,16^. In the proteoliposome studies, they used either immunostaining^9^ or applied functional assays^8,10^ to suggest which side of the interested proteins faces up. In the small native cell membrane vesicles, they found that the extracellular side of these vesicles faced toward the bulk solution by using enzymatic accessibility assays to cleave the overexpressed fluorescent proteins in their membranes. They suggested that their small membrane vesicles ruptured in a parachute manner. The difference between these studies could be explained by and also support the hypothesis by Tutus *et al*.^9^ that larger diameter vesicles (that is, those with diameters >1 μm) could rupture to form inside-out orientations while smaller diameter vesicles could rupture to form outside-out orientations. These may suggest that the size of the membrane vesicles could also be used to control the membrane orientations of supported plasma membranes.

## Materials and Methods

### Materials

Dithiothreitol (DTT) and paraformaldehyde (PFA) were purchased from Sigma-Aldrich (St. Louis, MO, USA). 3,3′-dilinoleyloxacarbocyanine perchlorate (Fast-DiO), and 1,1′-dilinoleyl-3,3,3′,3′-tetramethylindocarbocyanine perchlorate (Fast-DiI) were purchased from Invitrogen (Waltham, MA, USA). Rabbit anti-AQP3 C-terminus antibodies [PE/ATTO 594] (anti-cyto-AQP3) were purchased from Abnova (Taipei, Taiwan). Rabbit anti-AQP3 ecto-domain antibodies [FITC] (anti-ecto-AQP3) were purchased from FabGennix (Frisco, TX, USA).

### Hela cell culture and fluorescent GPMV formation

Hela cells were seeded in 6-cm cell culture dishes; cultured in a growth medium composed of 90% Dulbecco’s modified eagle medium (DMEM), 10% fetal bovine serum (FBS), 1.5 g/L sodium bicarbonate, 1 mM sodium pyruvate, and 100 unit/mL antibiotics; and incubated at 37 °C with 5% CO_2_. After growing for 1 day, the Hela cells were washed with phosphate buffered saline (PBS) buffer (10 mM NaH_2_PO_4_/Na_2_HPO_4_, 137 mM NaCl, 2.7 mM KCl, pH 7.4) three times and then incubated in 5 μg/mL of dye solution (Fast-DiO or Fast-DiI) at 4 °C for 10 min to stain the cell membranes. The cells were then washed three times with PBS buffer and GPMV buffer (10 mM HEPES, 150 mM NaCl, 2 mM CaCl_2_, pH 7.4) sequentially. Finally, the cells were incubated with 1 mL of vesiculant (25 mM PFA, 2 mM DTT in GPMV buffer supplemented with 4% protease inhibitor) at 37 °C for 1 hr to obtain GPMVs. The derived GPMVs were then collected and treated with dialysis at 4 °C overnight until used.

### Formation of the directly-deposited membrane

To form the directly-deposited membrane, the solution containing GPMVs was loaded into a PDMS well attached to mica and was allowed to sit for 1 hr. Two times the volume of DI-water was added to the GPMV-solution-containing well followed by 1 hr of incubation at room temperature to cause the GPMVs to rupture. Later, the well was washed extensively with GPMV buffer.

### Membrane blotting

Membranes were first formed on mica and then transferred to a glass coverslip pre-cleaned with argon plasma at a pressure of 100 Torr for 10 min. Before the directly-deposited membrane was blotted, the solution in the PDMS well was replaced by DI-water. 30 min later, the DI-water was replaced by PBS buffer, and the cleaned glass coverslip was immediately placed in contact with the original mica with the directly-deposited membrane. A pressure of 128 g/cm^2^ was then placed upon the two substrates from above for 20 min. After the membranes were transferred, the two substrates were separated in a water tank and rinsed with PBS buffer.

### Immunostaining of GPMVs and membranes

The GPMV suspension was stained by the antibodies with a final antibody concentration of 5 μg/mL for both rabbit anti-AQP3 C-terminus antibodies [PE/ATTO 594] (anti-cyto-AQP3) and rabbit anti-AQP3 ecto-domain antibodies [FITC] (anti-ecto-AQP3), after which it was incubated at room temperature for 1 hr. The stained GPMVs were washed three times by GPMV buffer using centrifugation at a speed of 100 g for 15 min. The stained GPMVs were then added to a well and observed under a microscope. The membranes were blocked in 5% BSA (in PBS) for 1 hr at room temperature before immunostaining. After blocking, each antibody was added into the well and incubated at room temperature for 1 hr with a final antibody concentration of 3 μg/mL for anti-cyto-AQP3 and 2 μg/mL for anti-ecto-AQP3. After the incubation, any unbound antibodies were washed away with PBS buffer, and the sample was observed under a microscope.

### Fluorescence microscopy and fluorescence recovery after photobleaching (FRAP)

Bright field and fluorescence images were captured by using an inverted microscope (Olympus IX81, Olympus, Japan) equipped with a charge-coupled-device camera (ORCA-R2, Hamamatsu, Japan) under a 10x or 20x object lens (UPLSAPO, Olympus). An X-cite mercury lamp was used for the fluorophore excitation. A 200 mW diode-pumped solid-state green laser module (Unice, Taiwan) was set at 532 nm and used to photobleach Fast-DiI and PE/ATTO594. A 200 mW diode-pumped solid-state blue laser module (Laserglow, Canada) was set at 473 nm and used to photobleach Fast-DiO and FITC. Images were processed with ImageJ (Wayne Rasband, National Institutes of Health (NIH)). The FRAP images were analyzed with Matlab software (MathWorks, Natick, MA, U.S.A.) using the algorithm we developed before^29^.

## Conclusions

Our results show the possibility of controlling the membrane orientation of a supported plasma membrane for the purpose of studying membrane proteins from either side of the cell plasma membrane. We used immunostaining of antibodies targeting either the ecto-domain or the cyto-domain of AQP3 to show that the GPMVs derived from chemical blebbing retained their cell membrane topologies and that the directly-deposited plasma membrane had its intracellular side facing toward the bulk solution. We further developed a blotting method to transfer a directly-deposited membrane to another support to reverse the membrane orientation. We used mica as the formation support and reduced the buffer ionic strength of the water layer between mica and the membrane to reduce the mica-membrane affinity before the blotting process. Decreasing the affinity of the membrane with the original mica support and maintaining/increasing the affinity with the blotting glass support led to effective blotting. The immunostaining results of the blotted membrane further confirmed that the membrane orientation or the membrane protein topology was reversed. The fluorescence recovery after photobleaching (FRAP) results showed that the membranes and proteins in both the directly-deposited and the blotted membrane platforms had fluidity. These results suggest that we are able to construct supported plasma membranes with controllable orientations to study membrane proteins from either side of the cell membrane.

## Supplementary information

Supplementary Information
