# Supplementary material for: Constructing Supported Cell Membranes with Controllable Orientation
Source: Sci Rep. 2019 Feb 26;9:2747. doi: 10.1038/s41598-019-39075-8 (PMC6391389; doi:10.1038/s41598-019-39075-8)
Supplement: Supplementary file 1 — Supplementary Information [file 41598_2019_39075_MOESM1_ESM.pdf]

Constructing Supported Cell Membranes with Controllable Orientation

Shao-Wei Lyu, Jou-Fang Wang and Ling Chao*,
Department of Chemical Engineering, National Taiwan University, Taipei, Taiwan
Email: lingchao@ntu.edu.tw

Supplementary Information

Topology of AQP3 and the immunogen sequences of the two used antibodies.

The two antibodies we used are known to target to the specific sequences of AQP3 in its cyto-domain and ecto-domain as shown in Figure S1. The blue highlighted region indicates the extracellular domain and the pink highlighted region indicates the intracellular domain of AQP3. The red and green boxes indicate the immunogen sequences of the two antibodies. The information is from the following two vendor websites. (http://www.abnova.com/products/products_detail.asp?catalog_id=PAB28888; https://fabgennix.com/Aquaporin-3-Antibody-FITC)

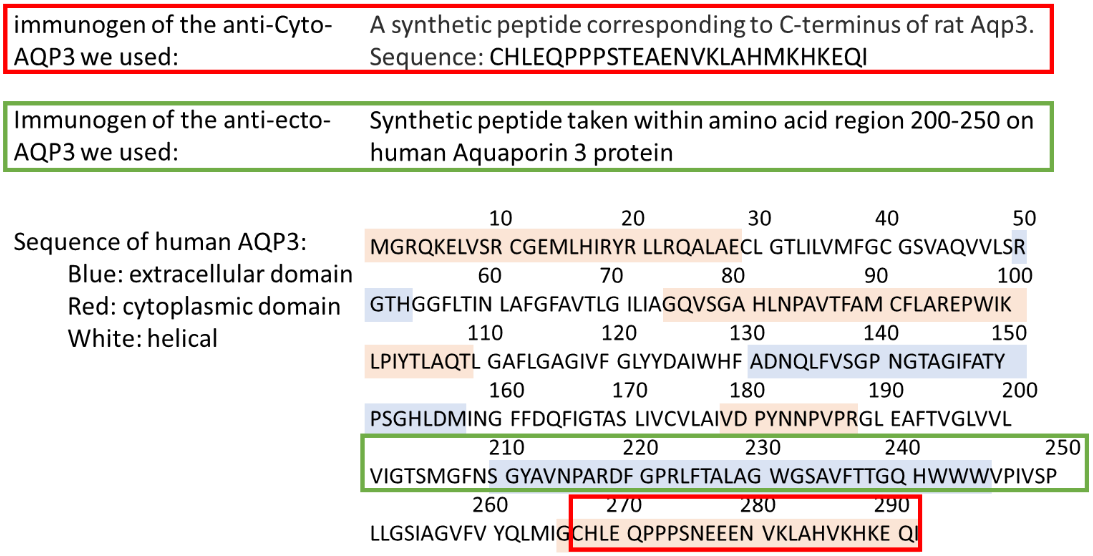

Figure S1. Topology of AQP3 and the immunogen sequences of the two used antibodies.

Binding of anti-aquaporin 3 to the pure 1,2-dioleoyl-sn-glycero-3-phosphocholine (DOPC) supported lipid bilayer and to the supported plasma membrane.

We have shown that the nonspecific binding of anti-cyto-AQP3 (anti-AQP3 C-terminus antibody [PE/ATTO 594]) to a typical supported lipid bilayer composed of pure DOPC is weak. Here we further examine whether the other antibody used in this study, anti-ecto-AQP3 (anti-AQP3 ecto-domain antibody [FITC]), would non-specifically bind to the lipid bilayer. We prepared the DOPC supported lipid bilayer and the blotted plasma membrane on glass supports. Both samples were blocked with 5% BSA (in PBS) for 1 hr at room temperature before the immunostaining. 2 μg/mL for anti-ecto-AQP3 was incubated with the samples at room temperature for 1 hr and washed away for the microscopic observation. The fluorescence intensity (FL) from the supported lipid bilayer composed of pure DOPC (Figure S2(a)) was much lower than the one from the blotted plasma membrane (Figure S2(b)), suggesting that the

nonspecific binding of the antibody to a typical supported lipid membrane is weak.

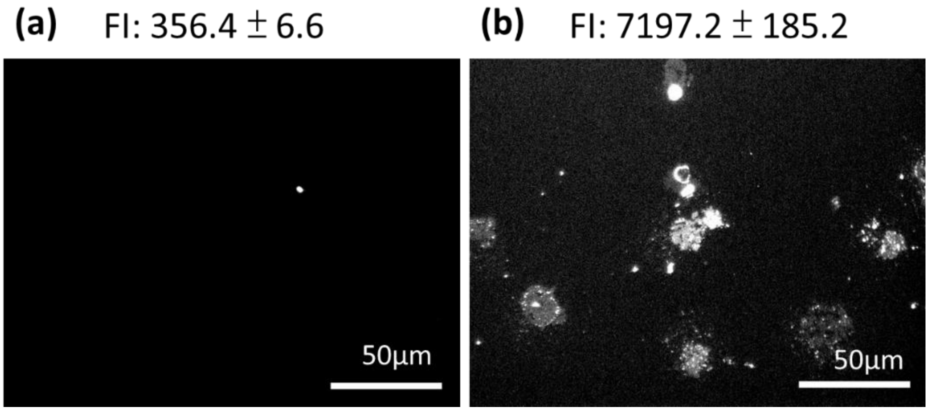

Figure S2. Fluorescence images of anti-AQP3 ecto-domain antibodies [FITC] (anti-ecto-AQP3) (a) in the pure DOPC supported lipid bilayer; (b) in the sample with blotted GPMV patches with the surrounding region blocked by BSA. FL: the averaged fluorescence intensity of the entire image.

Binding of labeled anti-glucose transporter GLUT1 antibody to the directly-deposited membrane.

The directly-deposited membrane on the mica were blocked with 2 mg/mL BSA for 1 hr at room temperature before the immunostaining. Rabbit anti-glucose transporter GLUT1 antibody (Abcam, USA) at 1:100 dilution was incubated with the sample at room temperature for 1 hr and washed away. The secondary antibody, Alexa Fluor™ 594 Goat anti-rabbit IgG (H+L) (Thermo Fisher Scientific, USA), at 1:100 dilution was later incubated with the sample at room temperature for another 1 hr and washed away for the microscopic observation. Figure S3 shows the positive immunostaining of anti-cyto-GLUT1 in the directly-deposited membrane, supporting that the intracellular side of the membrane faced toward the bulk solution.

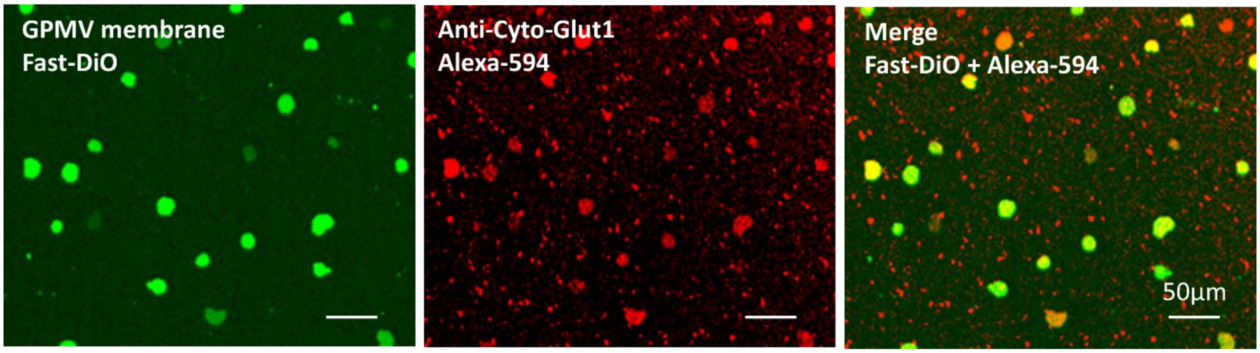

Figure S3. Fluorescence images of Fast-DiO labeled directly-deposited membrane patches (left) and the immunostaining result of a transmembrane protein, glucose transporter GLUT1 (middle). The right image is the merged image of the lipid probe (Fast-DiO) image and the antibody image.
